# Unravelling Sarcopenia in Chronic Kidney Disease: From Pathogenesis to Diagnosis and Therapeutics

**DOI:** 10.3390/diagnostics16071063

**Published:** 2026-04-01

**Authors:** Natalia G. Vallianou, Apostolos A. Evangelopoulos, Gerasimos Socrates Christodoulatos, Ioanna Tantsi, Nikos Mantouvalos, Dimitrios Chatzis, Theodora Stratigou, Eleni V. Geladari, Kyriaki Constantinou, Alexandros Tousis, Dimitris C. Kounatidis

**Affiliations:** 1 First Department of Internal Medicine, Sismanogleio General Hospital, 15126 Athens, Greece; ioannatantsi@gmail.com (I.T.); nickmantouvalos@gmail.com (N.M.); dimitris.chatzis2@gmail.com (D.C.); 2 Medical School, National and Kapodistrian University of Athens, 11527 Athens, Greece; apostolos.evangelopoulos.nak@gmail.com; 3 Department of Microbiology, Sismanogleio General Hospital, 15126 Athens, Greece; gerchristod82@hotmail.com; 4 Diabetes and Endocrinology Department, Evangelismos General Hospital, 10676 Athens, Greece; theodorastratigou@yahoo.gr; 5 Third Department of Internal Medicine, Evangelismos General Hospital, 10676 Athens, Greece; elgeladari@gmail.com; 6 Second Department of Surgery, Sismanogleio General Hospital, 15126 Athens, Greece; drconstantinou@hotmail.com; 7 Department of Cardiology, University Hospital of Patras, 26504 Patra, Greece; alextousis21@gmail.com; 8 Diabetes Center, First Propaedeutic Department of Internal Medicine, Medical School, National and Kapodistrian University of Athens, Laiko General Hospital, 11527 Athens, Greece; dimitriskounatidis82@outlook.com

**Keywords:** sarcopenia, chronic kidney disease, mortality, quality of life, diagnosis, potential interventions

## Abstract

Chronic kidney disease (CKD) is on the rise, with sarcopenia accompanying CKD in an estimated 25% of patients, featuring as a potentially debilitating issue that should not be overlooked. Sarcopenia, characterized by a loss of skeletal muscle mass and strength, is multifactorial. The aging process, uremic toxins, systemic inflammation, oxidative stress, gut dysbiosis, hormonal dysregulation, dietary deficits, and even air pollution are among the major parameters being implicated in sarcopenia among patients with CKD. Additionally, the existence of various comorbidities, such as type 2 diabetes mellitus (T2DM), depression, and cardiovascular diseases (CVD), also contribute to the chronic low-grade inflammation associated with skeletal muscle inflammation and atrophy. The purpose of this review is to delve into the complex interplay of multiple factors being involved in the pathogenesis of sarcopenia in patients with CKD. Moreover, we aim to shed light upon nutritional aspects that could delay the development and progression of sarcopenia among patients with CKD. To address vitamin D deficiency, micronutrients and macronutrients together with physical activity remain the cornerstone of delaying the progression of sarcopenia in this sub-population. Additionally, experimental drugs exhibiting therapeutic potential are also being discussed. As sarcopenia and quality of life are interconnected, the timely recognition of sarcopenia, together with nutritional and therapeutic interventions, is of the utmost importance in our crusade for a better quality of life (QoL) in patients with CKD.

## 1. Introduction

According to the 2024 definition by KDIGO (Kidney Disease: Improving Global Outcomes), chronic kidney disease (CKD) is defined as the presence of kidney damage, for at least 3 months, that impacts health [1]. The two most commonly used markers suggestive of CKD are a reduction in the estimated glomerular filtration rate (eGFR) of below 60 mL/min/1.73 m^2^ (category G3a-G5), and albuminuria. The risk of death is increased among patients with eGFR < 60 mL/min/1.73 m^2^ and mild albuminuria. However, it is multiplied in cases of severe albuminuria and even lower eGFR [1].

In 2023, an estimated 788 million people aged > 20 years old had CKD globally, whereas in 1990 there were approximately 378 million. Additionally, in 2023, CKD was the ninth leading cause of death worldwide [2]. The burden of CKD has been on the rise due to the aging population. However, it is noteworthy that comorbidities like obesity, type 2 diabetes mellitus (T2DM), and hypertension are major contributing factors [2,3]. CKD has been associated with the presence of sarcopenia in 11% to 30% of patients, with this proportion being worse among patients with end-stage renal disease (ESRD) on dialysis [4,5].

Sarcopenia has been related to a distorted quality of life (QoL) due to increased falls, fractures, and subsequently higher rates of hospitalization and overall mortality [5,6,7]. The purpose of this review is to elaborate upon the complex pathogenetic mechanisms behind sarcopenia among patients with CKD. Apart from pathogenetic pathways, we aim to further expand our understanding of the significance of sarcopenia in clinical practice. Preventive measures and nutritional interventions that could help delay the progression of sarcopenia will be thoroughly described. Moreover, experimental therapeutic strategies will also be discussed.

## 2. Search Methodology

On 5 January 2026, we searched the PubMed database using the terms “sarcopenia and CKD” for papers published during the past five years. This search yielded 346 manuscripts, among which 58 were excluded due to the following reasons: (1) they dealt exclusively with patients on hemodialysis and peritoneal dialysis, (2) they were associated with cardiovascular and pulmonary complications, (3) they were referring to cancer patients, and (4) they were written in a foreign language. Thus, a total of 282 publications were finally included for the purpose of this review. However, we acknowledge that not all of these articles can be covered in the context of this review.

## 3. Pathogenetic Factors and Molecular Implications

**Figure 1:**
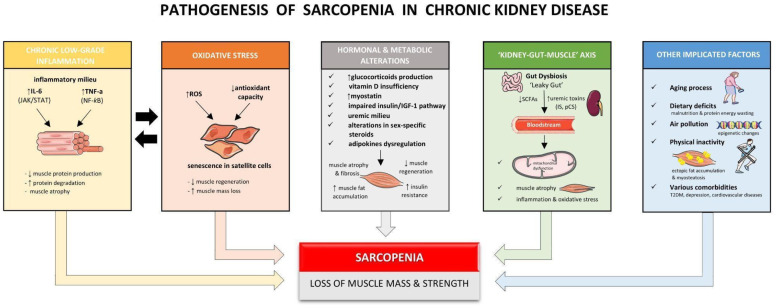
Pathophysiological factors involved in the pathogenesis of sarcopenia among patients with chronic kidney disease (CKD). Figures adapted from Servier Medical Art (https://smart.servier.com), licensed under CC BY 4.0 (https://creativecommons.org/licenses/by/4.0/, accessed on 1 March 2026). Abbreviations: IGF-1: Insulin-like growth factor 1; IL: Interleukin; IS: Indoxyl sulfate; JAK/STAT: Janus Kinase/Signal Transducers and Activators of Transcription proteins; NF-*k*B: Nuclear factor kappa B; pCS: p-Cresyl sulfate; ROS: Reactive oxygen species; SCFAS: Short-chain fatty acids; T2DM: Type 2 diabetes mellitus; TNF-a: Tumor Necrosis Factor alpha.

Sarcopenia is characterized by reduced skeletal muscle mass and strength with severe consequences, such as falls and fractures. It has been associated with increased morbidity and mortality. Sarcopenia is the result of impaired skeletal muscle protein synthesis, an enhancement in muscle protein degradation, or both [8,9,10]. This imbalance between the rate of protein synthesis and degradation in skeletal muscles has been attributed to various factors, such as chronic low-grade inflammation, increased oxidative stress, hormonal and metabolic alterations, nutritional parameters, and gut dysbiosis [11,12,13,14]. Figure 1 depicts several factors implicated in the pathogenesis of sarcopenia among patients with CKD.

### 3.1. Chronic Low-Grade Inflammation

CKD has been related to a state of chronic low-grade inflammation, as evidenced by the increased serum levels of the pro-inflammatory cytokines Interleukin-6 (IL-6) and Tumor Necrosis Factor alpha (TNF-a). TNF-a in turn, activates Nuclear Factor kappa B (NF-*k*B), leading to muscle protein degradation. IL-6 induces muscle atrophy by means of the Janus Kinase/Signal Transducers and Activators of Transcription protein (JAK/STAT) pathway [12,13,14,15]. More specifically, NF-*k*B is a key regulator of the Nucleotide-binding domain-like receptor protein 3 (NLRP3) inflammasome, thus controlling the release of the pro-inflammatory cytokines IL-1β and IL-18 [16]. Additionally, IL-6, by binding to skeletal muscle receptors, activates JAK, which subsequently phosphorylates STAT. Phosphorylated STAT moves and is finally located in the nucleus to further regulate gene transcription, like atrophy-related genes [17,18]. Overall, this inflammatory milieu sustains a vicious cycle leading to impaired muscle protein production, increased protein degradation, and the acceleration of muscle atrophy [12,13,14,15,16,17,18].

### 3.2. Oxidative Stress

Oxidative stress refers to the imbalance in the redox cellular capacity toward the increased production of reactive oxygen species (ROS) and reactive nitrogen species (RNS). Mitochondria are the main organelle associated with the production of ROS. Increased ROS production by mitochondria may lead to DNA damage via lipid peroxidation.

The latter has been suggested to be a contributing factor in shaping toxic compounds to muscle cells, such as 4-hydroxynonenal. This muscle-toxic substance may further exacerbate muscle damage [19]. Thus, mitochondrial dysfunction results in excess ROS production and reduced antioxidant cellular capacity [19]. Moreover, increased ROS production has been associated with senescence in satellite cells (SCs). SCs are cells located between the sarcolemma and the basal membrane of myofibers. They possess the capacity to induce muscle regeneration. However, excess production of ROS may disturb the ability of the SCs to promote muscle regeneration, ultimately resulting in muscle mass loss [20,21]. Oxidative stress and chronic low-grade inflammation are intricately interrelated. The disturbed redox capacity of the cells may induce a state of chronic low-grade inflammation, while inflammation per se may increase the production of ROS. The production of ROS is augmented in an attempt to defend cells from various pathogen-associated molecular patterns (PAMPs), such as the lipopolysaccharides (LPS) of Gram-negative bacteria. Notably, ROS possess the capacity to activate NF-*k*B, which, in turn, may further exacerbate inflammation. Thus, oxidative stress and inflammation are interconnected in a bidirectional manner [20,21,22].

### 3.3. Hormonal and Metabolic Changes

Hormonal changes that are commonly seen among patients with CKD are secondary hyperparathyroidism, vitamin D insufficiency, insulin resistance with impaired insulin-like growth factor 1 (IGF-1) secretion, increased levels of glucocorticoids, disturbances in age- and gender-specific steroids, and thyroid gland hormone dysfunction. In particular, vitamin D insufficiency, which has been associated with secondary hyperparathyroidism, has been implicated in muscle metabolism [11,12,13].

Vitamin D insufficiency, a conventional finding in patients with CKD, has been demonstrated to be related to type II muscle fiber atrophy, fat accumulation in muscles, and fibrosis. Interestingly, vitamin D affects various myokines and osteokines, like IGF-1, myostatin, follistatin, and fibroblast growth factor (FGF), thus intervening in muscle cell synthesis [23]. Myostatin, which belongs to the transforming growth factor β (TGF-β) superfamily, has been documented to be elevated in patients with CKD, More specifically, myostatin has been implicated in suppressing muscle growth. Serum myostatin levels are increased in patients with CKD due to an enhancement in its synthesis by muscles combined with its reduced renal clearance. The binding of myostatin to activin receptors in muscle cells result in the activation of the STAT3 signaling pathway, leading to increases in atrogene expression [24]. Atrogenes are atrophy-related genes encoding proteolytic enzymes like atrogin-1 and muscle RING Finger 1 (MuRF-1). Myostatin has been demonstrated to affect SCs, thus contributing to the state of SC senescence and impaired muscle regeneration in CKD [11,12,13,24]. Additionally, myostatin has been associated with insulin resistance in muscles [25].

There is mounting evidence supporting the notion that the insulin/IGF-1 pathway is impaired among patients with CKD. This impairment could be attributed to a variety of factors, such as inflammation, increased glucocorticoid production, and metabolic acidosis. The insulin/IGF-1 pathway via phosphoinositide-3-kinase/protein kinase B/mammalian target of rapamycin (PI3K/Akt/mTOR) kinase activity has been suggested to be a hallmark of muscle degeneration and atrophy in CKD [26,27]. Notably, glucocorticoids have been reported to enhance the expression of atrogenes, such as myostatin and MuRF-1. Myostatin and MuRF-1 have binding sites for both glucocorticoids and forkhead box protein O (FOXO) transcription factors, thus pointing toward a synergistic action of these transcription factors [11,12,13]. Regarding age- and gender-specific steroids, it is noteworthy that in postmenopausal women there is an abrupt disturbance in the secretion of steroids, whereas in men, testosterone gradually decreases in a more accelerated manner after the age of 80. Nevertheless, together with the aging process and steroid production alterations, sarcopenia rates are rising, especially in the context of CKD [5].

Furthermore, the uremic milieu, which has been mainly associated with increased levels of protein-bound uremic toxins such as indole sulfate (IS) and p-cresyl sulfate (pCS), also accounts for muscle atrophy among patients with CKD. It has been suggested that in late stages of CKD, IS, pCS, and secondary hyperparathyroidism further exacerbate sarcopenia [5,11,12,13]. Interestingly, serum IS levels have been reported to be inversely related to handgrip strength [28,29]. Moreover, thyroid gland dysfunction occurs much more often among patients with CKD. Indeed, subclinical hypothyroidism and low T3 syndrome are the most frequently encountered thyroid gland disorders in these patients [23,29,30]. It seems likely that thyroid function and CKD share a bidirectional relationship. There is growing evidence advocating that, apart from the subclinical thyroid syndromes associated with CKD, thyroid hormones play a crucial role in kidney function and structure as well [24,29,30].

Additionally, leptin, a hormone secreted mainly from adipose tissue, has been postulated to be lower among patients with CKD. Notably, leptin deficiency or resistance has been associated with sarcopenia. In particular, leptin facilitates the oxidation of free fatty acids, preventing cells from lipotoxicity, while increasing mitochondrial capacity and promoting muscle mass [24,31]. Therefore, its deficiency or resistance may lead to the development of sarcopenia. Ghrelin, a hormone secreted from the stomach, has the potential to increase appetite and nutrient intake while regulating the secretion of growth hormone (GH). Serum ghrelin levels are decreased among patients with CKD, and this reduction could play a pivotal role in sarcopenia seen in these patients [24,32]. However, more large-scale studies are needed to further evaluate the significance of the aforementioned hormonal changes in the development of sarcopenia in CKD.

### 3.4. Epigenetic Changes

Epigenetics refers to changes in DNA methylation, histone modification, and RNA-based regulatory pathways. The hypermethylation or hypomethylation of DNA and alterations in chromatin organization and non-coding RNA mechanisms are all considered epigenetic changes [33]. At the skeletal muscle level, epigenetics has focused on myofibers and SC reprogramming rather than changes in DNA sequencing. The aging skeletal muscles undergo age-related methylation remodeling toward a state of augmented muscle breakdown. This catabolic state has been the cornerstone of sarcopenia among CKD patients and the aging population [33]. Denervation has been demonstrated as a significant upstream driver of sarcopenia. Notably, after denervation, histone deacetylase 4 (HDAC4) translocates to the myofiber nuclei, where it remodels chromatin organization toward the expression of catabolic genes [34]. Very recently, 7379 differential methylation positions (DMPs) were found among patients suffering from muscle weakness who were admitted to an ICU when compared to controls [35]. Furthermore, Wei et al. followed a multi-omics approach and Mendelian randomization study and proposed that air pollution, through epigenetic changes, may be implicated in sarcopenia development among patients with cardiovascular–kidney–metabolic syndrome (CKM). More specifically, they found 13 air pollution genes in blood, among which 11 were cross confirmed with tissue validation. They concluded that air pollution might drive sarcopenia in CKM and proposed various genes and methylation sites that could link these two conditions [36]. This study may pave the way for more thoroughly explored associations between genetic and epigenetic factors regarding sarcopenia in CKD. Moreover, findings from the Health and Retirement Study have shown that physical activity is linked to reductions in epigenetic aging. This remarkable finding relating to accumulated and recent physical activity points toward the beneficial effects of physical activity in terms of halting or delaying epigenetic aging [37]. The study of age-related epigenetic changes regarding sarcopenia, especially among patients with CKD, is still in its infancy. Nevertheless, this field is expected to thrive in the near future.

### 3.5. Nutritional Parameters

Malnutrition and protein energy wasting (PEW) have both been implicated in the pathogenesis of sarcopenia in CKD. Diets poor in micronutrients and macronutrients may result in deficient muscle protein synthesis in patients with CKD [38,39]. Essential amino acids, i.e., amino acids necessary for protein synthesis that should be obtained from food as the human body cannot synthesize them, are of utmost importance in this context. Among them, the branched-chain amino acid (BCAA) leukine, which acts via the mTOR signaling pathway, is especially helpful in muscle protein synthesis [40]. In addition to essential amino acids, other nutritional parameters, such as zinc, selenium, thiamin, and vitamin D deficiency, have also been more frequently observed among patients with CKD [41,42]. Regarding macronutrients, the amount and type of protein intake still remains an issue of ongoing debate. The optimal proportion of protein intake by patients with CKD has been suggested to vary according to age and stage of CKD. Additionally, animal- and plant-based protein intake has been extensively studied recently [43,44].

### 3.6. Physical Inactivity

Physical inactivity contributes to decreased muscle protein synthesis as well as increased muscle protein degradation among patients with CKD. This sub-population usually exhibits reduced physical activity due to concomitant anemia, chronic fatigue, and the aging process. Moreover, other comorbidities, such as T2DM, hypertension, obesity, and depression may also account for physical inactivity among patients with CKD [44,45]. Yang et al. demonstrated an association between decreased physical activity and sarcopenia; they also documented that physical activity protects against sarcopenia in patients with CKD [45]. Notably, sarcopenic obesity (SO) is characterized histologically by the presence of myosteatosis due to ectopic fat accumulation in the muscles. SO is indicative of the complex interplay between physical inactivity, malnutrition, and chronic low-grade inflammation. Overall, lifestyle seems to be a major determinant of the presence and severity of sarcopenia in patients with CKD [46,47,48].

### 3.7. Gut Dysbiosis

The term “gut dysbiosis” refers to the imbalance between the human gut microbiota and the human body. Under normal circumstances, there is homeostasis between the gut microorganisms inhabiting the human intestines and the human body. However, when this balance is interrupted, the phenomenon of gut dysbiosis occurs [49,50]. Gut dysbiosis has been associated with the disruption of the intestinal barrier, mainly attributed to the disruption of tight junctions (TJs). Distorted TJs permit the entry of various PAMPs, like the LPS of Gram-negative bacteria from the gut lumen into systemic circulation. This so-called “leaky gut” may lead to a release of pro-inflammatory cytokines, such as IL-6 and TNF-a, thus sustaining a state of chronic low-grade inflammation [50,51]. It is noteworthy that in patients with sarcopenia and CKD, the term “kidney–gut–muscle axis” has been proposed in order to better describe the consequences of gut dysbiosis and chronic low-grade inflammation in the context of a uremic milieu [52].

Regarding the kidney–gut–muscle axis in patients with CKD, the blood levels of IS and pCS are increased. The enhanced production of IS and pCS by *Enterobacterales* in the gut, together with the impaired intestinal barrier that allows for their release in the blood, account for the elevated blood concentrations of IS and pCS [14]. As already mentioned, increased IS and pCS levels have been associated with muscle atrophy, as confirmed by increased atrophy markers such as myostatin and atrogin-1. Furthermore, decreased muscle mass and muscle atrophy have been related to insulin resistance and myosteatosis. Notably, IS has been found to enhance glycolysis via the nuclear factor 2 (Nrf-2) (erythroid-2-related factor-2) signaling pathway, while downregulating the tricarboxyl acid (TCA) cycle. Together, these actions result in reductions in ATP due to mitochondrial dysfunction [14].

The production of short chain fatty acids (SCFAs) by the gut microbiota has been suggested to be decreased in patients with CKD. Interestingly, SCFAs, like butyrate and acetate, have been shown to possess anti-inflammatory and anti-oxidative properties, while promoting the normal function of TJs. Thereby, their reductions have been linked to the phenomena of “leaky gut”, inflammation, and oxidative stress, which are all well-known factors contributing to sarcopenia in CKD [51,52].

## 4. Diagnosis

The European Working Group on Sarcopenia in Older People 2 (EWGSOP2) issued the criteria for the definition of sarcopenia in 2019. These criteria focus on (1) decreased muscle strength as an essential component of sarcopenia, (2) lower muscle quantity or quality, and (3) poor physical performance indicative of severe sarcopenia. The first EWGSOP assembly took place in 2010, with the EWGSOP2 in 2018 attempting to update the EWGSOP criteria according to novel findings and the latest technologic advances regarding sarcopenia [53].

Decreased muscle strength is evaluated by handgrip strength and the chair stand test. A handgrip strength <27 kg for males and <16 kg for females, as assessed by a dynamometer, and a chair stand test result of >15 s over five tests indicate “probable sarcopenia”. Patients found to have probable sarcopenia should be further evaluated for muscle quantity or quality by dual energy X-ray absorptiometry (DEXA) or bioelectrical impedance analysis (BIA) to confirm sarcopenia. Muscle quantity/quality is performed using appendicular skeletal muscle mass (ASMM), with ASMM < 20 kg for males and <15 kg for females used as cut-offs. The use of the appendicular skeletal muscle index (ASMI), which is the value of ASMM divided by square height [ASMM/(Height)^2^], is another marker for evaluating muscle quantity/quality. ASMI <7 kg/m^2^ for males and <5.5 kg/m^2^ for females are considered cut-off values. When patients with probable sarcopenia meet the ASMM and/or ASMI values below the abovementioned cut-offs, then they are considered to have sarcopenia.

Furthermore, patients are classified as having severe sarcopenia when they have (i) a 6 m gait speed ≤ 0.8 m/s; (ii) a Short Physical Performance Battery (SFPB) value ≤8 points; (iii) and a timed up and go (TUG) test result ≥20 s, i.e., the time to stand from a chair, walk for 3 m, turn around, walk back, and sit on the same chair. It is noteworthy that apart from DEXA and BIA, computed tomography (CT) and magnetic resonance imaging (MRI) are suggested to be the gold standard for measuring muscle quantity/quality. However, MRI and CT may not be easily accessible, are more expensive, need more sophisticated equipment, and have been associated with more radiation exposure in the case of CT [54,55]. Ultrasound has also been a relatively low-cost and accurate technique for assessing sarcopenia in patients with CKD [56]. Muscle thickness and echo intensity together with the ability to perform assessment at the bedside are the main advantages of ultrasound when evaluating sarcopenia [56].

Table 1 depicts the definitions of probable sarcopenia, definitive sarcopenia, and severe sarcopenia according to the EWGSOP2 criteria. Notably, other criteria also exist, such as the Asian Working Group for Sarcopenia (AWGS) criteria, which are slightly different from those of the EWGSOP2 based on differences due to race [13,14].

Another diagnostic tool for sarcopenia in patients with CKD is the Strength, Assistance with walking, Rising from the chair, Climbing stairs and Falls (SARC-F) questionnaire. This questionnaire is briefly described in Table 2. A SARC-F Score ≤3 has been associated with a low probability of sarcopenia, whereas a SARC-F Score of ≥4 has been linked to a significant risk of sarcopenia [57,58]. The SARC-F Score has been found to effectively predict sarcopenia among patients with ESRD [57,58].

It is noteworthy that another candidate for mortality among patients with CKD is respiratory sarcopenia. Respiratory sarcopenia is defined by decreased respiratory muscle strength as evidenced by a low peak expiratory flow rate (PEFR) together with low respiratory muscle mass. It results in reduced pulmonary function and shortness of breath. Indeed, Yao et al. recently found respiratory sarcopenia to be an independent risk factor of all-cause mortality among patients with CKD. In their study, they enrolled 1300 patients with CKD from the CHARLS Study and 1346 patients from the US Health and Retirement Study. They reported that respiratory sarcopenia, as defined by measurements such as peak expiratory flow rate and ASMI was associated with all-cause mortality, while the reversion of respiratory sarcopenia was related to a lower risk of mortality [59]. Zhang et al., in their systematic review and meta-analysis of 6217 patients with CKD, documented that a slower gait speed correlates with higher mortality rates among patients with CKD [60].

Notably, the EWGSOP2 criteria and the SARC-F Score provide information about sarcopenia based on clinical grounds. However, biomarkers that exhibit the ability to describe sarcopenia are lacking. This is especially true for patients with CKD and sarcopenia. Interestingly, patients with sarcopenia and CKD comprise a sub-population distinct from the general population in terms of age, sex, race, fluid overload, and muscle mass. Therefore, it is rather difficult to find an ideal biomarker in this context [61,62].

The triglyceride–glucose (TyG) index, as assessed by the formula proposed by Guerrero-Romero et al., has been evaluated for the diagnosis and severity of sarcopenia among patients with CKD [63]. More specifically, the increased TyG index has been demonstrated to be correlated with sarcopenia in patients with CKD. This finding was documented in the NHANES 2011–2018 cohort in the US and the NHANES 2018–2023 cohort in China, having enrolled 827 patients with CKD in the US and 1038 patients with CKD in China, respectively [64]. This association may be indicative of the insulin resistance signaling pathway being implicated in the development of sarcopenia in CKD. It is noteworthy that an increased TyG index may reflect skeletal muscle dysfunction due to lipotoxicity (elevated triglycerides) and glucotoxicity (elevated FPG).

For the determination of the TyG index, the formula requires only fasting triglyceride and fasting plasma glucose (FPG) levels. Therefore, the TyG index may serve as a very easily performed, convenient, and inexpensive tool for evaluating sarcopenia among patients with CKD [64]. Very recently, Zhao et al. developed a nomogram and machine learning model to predict sarcopenia among patients with CKD. They validated this machine learning program based on the China Health and Retirement Longitudinal Study (CHARLS) data. They concluded that this machine learning model may be an effective tool for evaluating patients with CKD at high risk for developing sarcopenia [65]. Interestingly, nomograms and machine learning programs may counteract the drawbacks of simple biomarkers, which have already been mentioned before.

Undoubtedly, there is an unmet need for biomarkers predicting the existence and severity of sarcopenia together with overall mortality among patients with CKD. Recently, the serum creatinine to cystatin C ratio (CCR) has been proposed as a potential biomarker. Serum creatinine and serum cystatin C are both GFR biomarkers. However, unlike cystatin C, creatinine is also influenced by body weight and muscle mass. Cystatin C is a protein produced ubiquitously from cells and excreted from the kidneys, largely unaffected by muscle mass [66]. Zheng et al. performed a meta-analysis that demonstrated that CCR is a valuable tool in evaluating muscle mass and quality in patients with CKD, whether on dialysis or not. In particular, they reported that a higher CCR has been associated with a lower mortality rate among patients with CKD, irrespective of their dependence on dialysis [67]. They also documented that CCR is an effective and simple tool for assessing sarcopenia in hospitalized patients, which could add prognostic information regarding mortality rates [68].

Notably, the difference between estimated GFR (eGFR) based on cystatin C levels and eGFR based on serum creatinine levels, known as eGFRdiff, has also been reported as a surrogate biomarker regarding falls, frailty and CKD [69,70]. Furthermore, eGFRdiff and Growth Differentiation Factor-15 (GDF-15) were shown to be associated with mortality and CKD progression among 638 patients with DM [71]. GDF-15 belongs to the transforming growth factor beta superfamily and has been linked to inflammation, as it is a stress-inducible cytokine. Nowadays, GDF-15 is suggested to be a potential cardiometabolic biomarker, as its enhancement has been related to cardiometabolic disorders such as T2DM, obesity, atherosclerosis, heart failure and CKD. Although its precise role has not been fully elucidated, there is accumulating evidence advocating its role in metabolic inflammation [72,73].

Collectively, the aforementioned data are suggestive of a relationship between markers of sarcopenia and mortality among patients with CKD. However, further large-scale studies are needed to confirm whether the reversal of severe sarcopenia is linked to reduced mortality rates in this population.

## 5. The Importance of Nutritional Interventions and Physical Exercise

In order to prevent the development and progression of sarcopenia in patients with CKD, nutritional interventions, mainly pursuing an anti-inflammatory diet, have been suggested. An anti-inflammatory diet comprises food items such as fruits, vegetables, whole grains, legumes, nuts, oily fish, and plant-based proteins. A plant-based diet per se possesses anti-inflammatory properties. However, there are concerns that a plant-based diet may be related to PEW and hyperkalemia. Nevertheless, a well-balanced plant-based diet may actually result in the amelioration of PEW and prevention of hyperkalemia [74,75]. Indeed, a plant-based diet rich in fruits, vegetables, micronutrients and phytochemicals has been advocated to be beneficial regarding sarcopenia in patients with CKD [76,77].

Nutraceutical-induced Nrf-2 activation with grape polyphenols; resveratrol; phenolics in green tea, such as epigallocatechin-3-gallate; ursolic acid, such as in rosemary, blueberry, and apple peels; and omega-3 should be further studied as part of various nutritional interventions in the near future [78]. Moreover, vitamin D must be substituted in patients with CKD [78,79]. Moreover, according to the KDIGO guidelines, protein intake in patients on dialysis should be between 1.0 and 1.2 g/kg daily, whereas in patients with CKD stages 3–5, a protein intake of 0.8 g/kg daily is recommended [78,79]. Apart from the regular assessment of dietary intake in patients with CKD, a healthy diet with vitamin-dense foods is considered to be most efficacious in this sub-population [80,81,82]. Additionally, physical activity with aerobic exercise, such as cycling or walking, and resistance exercise to improve muscle mass and strength are of utmost importance [5]. The regulation of insulin release, mitigation of endoplasmic reticulum stress (ERS), and amelioration of endothelial dysfunction may be attributed to regular, moderate exercise. These favorable outcomes together with improvement in QoL may be accomplished through physical activity, provided this activity is moderate and not intense and exaggerated [5].

## 6. Potential Therapeutics

Nowadays, there is increasing interest in the prevention and treatment modalities regarding sarcopenia in CKD. In an animal model of adenine-induced CKD, Iwamoto et al. reported that lactoferrin administration resulted in the amelioration of sarcopenia in mice with CKD. More specifically, lactoferrin is an iron-binding glycoprotein that has been implicated in the activation of the mTOR pathway, autophagy, and BCAA metabolism. By mitigating the production of microbiota-derived uremic toxins, lactoferrin led to the attenuation of uremic sarcopenia, most probably via the kidney–gut–muscle axis [83]. In another mouse model of CKD, Akhter et al. recently demonstrated that the administration of the myostatin antisense oligonucleotide (ASO) KMM001 had beneficial effects regarding muscle atrophy in CKD. In particular, they documented that the subcutaneous administration of myostatin-ASO resulted in the downregulation of myostatin, atrogin-1, and MURF-1 genes, thus mitigating muscle protein catabolism. Notably, the skeletal muscle levels of Collagen-I were also decreased, a finding that was suggestive of a reduction in the rate of muscle fibrosis. Overall, their data pointed toward encouraging effects of this myoastatin-ASO [84]. In another study, Transcriptional Intermediary Factor-1γ (TIFγ), which is known to be involved in the TGF-β1 pathway, was found to improve muscle performance in a mouse model of diabetic nephropathy. Interestingly, treatment with TIF-1γ resulted in increased muscle and blood irisin concentrations as well as in decreased atrophy-related gene expression [85].

Regarding human studies, AST-120, an oral absorbent of uremic toxins, has been suggested to be effective in the RECOVERY Study that enrolled 150 participants. The RECOVERY Study, which lasted for 48 weeks, was a randomized controlled trial (RCT) that showed a modest improvement in gait speed change and QoL [86]. Furthermore, selective peroxisome proliferator-activated receptor-α modulator (SPPARMα) has been shown to improve hypertriglyceridemia and muscle quality in patients with chronic kidney disease. Indeed, Mae et al., while studying 245 patients with hypertriglyceridemia and CKD, documented that those who were administered SPPARMα for hypertriglyceridemia exhibited amelioration in muscle quality, as assessed by BMI, ASMI and phase angle [87].

**Figure 2:**
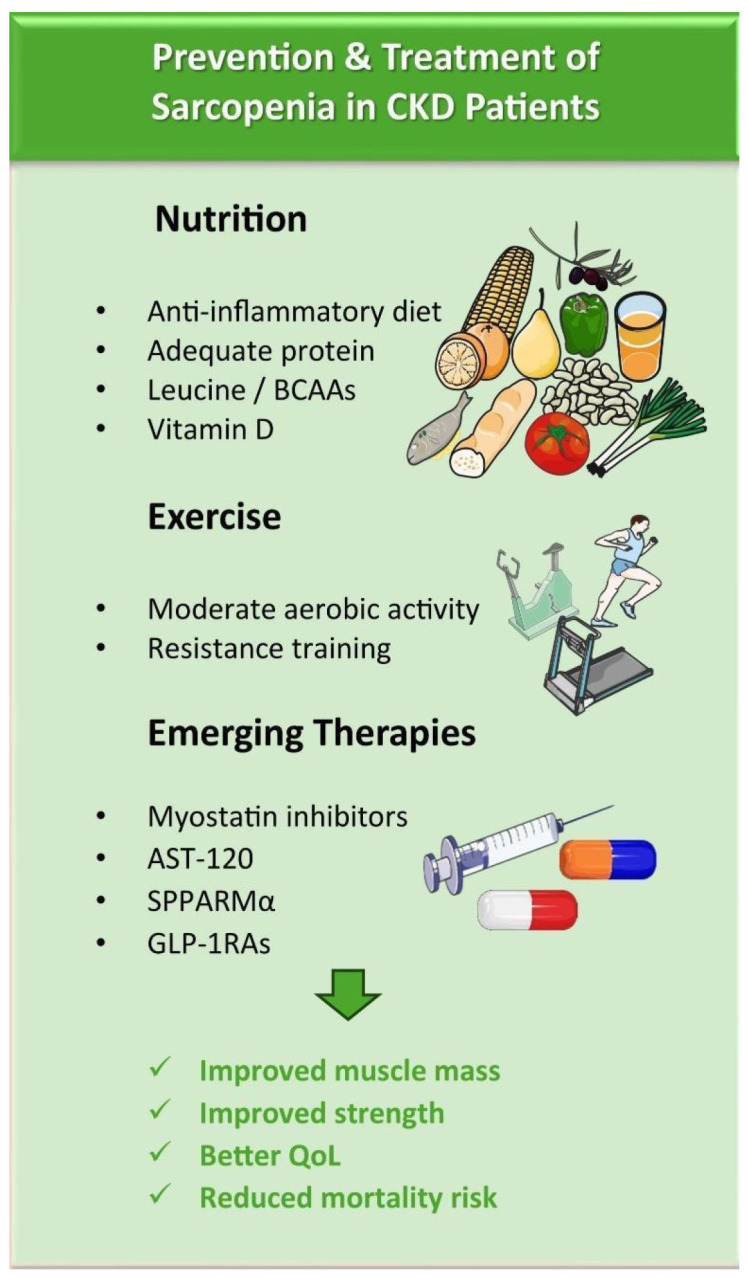
**Prevention and treatment of sarcopenia in CKD patients.** Figures adapted from Servier Medical Art (https://smart.servier.com), licensed under CC BY 4.0 (https://creativecommons.org/licenses/by/4.0/, accessed on 1 March 2026). Abbreviations: BCAAs: Branched-Chain Amino Acids; CKD: Chronic Kidney Disease; GLP-1RAs: Glucagon-Like Peptide 1 Receptor Agonists; QoL: Quality of Life; SPPARMα: Selective Peroxisome Proliferator-Activated Receptor-α Modulator.

Glucagon-like Receptor Agonists 1 (GLP-1RAs) and dual GLP-1RAs/GIP (Glucose-Dependent Insulinotropic Polypeptide) have been associated with anti-inflammatory and anti-oxidative properties in addition to their effects in terms of glycemic and weight control. Based upon their aforementioned features, GLP-1RAs have been documented to contribute to weight loss [88]. However, although there are preliminary results from the STEP1 Study (Semaglutide Treatment Effect in People with Obesity) and the SURPASS-3 and SURPASS-5 studies with tirzepatide suggesting a role for semaglutide and tirzepatide in preserving lean mass, there is still much uncertainty about their effects on sarcopenia in CKD [89,90,91,92]. The STEP1, SURPASS-3, and SURPASS-5 studies involved measurements such as MRI and DEXA regarding muscle status. Nevertheless, further studies are eagerly anticipated to shed light upon this issue, especially among patients with CKD. Figure 2 summarizes potential therapeutics and strategies to prevent the development and progression of sarcopenia in patients with CKD.

## 7. Future Perspectives

Despite the fact that there are several definitions of sarcopenia, there is consensus that sarcopenia poses a serious health problem among patients with CKD. As sarcopenia has been associated with a distorted QoL and increased mortality in patients with CKD, this clinical entity should not be overlooked [91,92,93]. Indeed, there is growing interest regarding the timely diagnosis of sarcopenia. For example, the suggested use of multiparametric quantitative MRI is a non-invasive method offering the opportunity for the early detection of anatomical muscle pathology before the establishment of muscle dysfunction [94]. In this context, the integration of machine learning and artificial intelligence (AI) in general could be very helpful in the early diagnosis of sarcopenia in patients with CKD [95,96,97]. Moreover, sophisticated technical equipment, such as the use of multi-omics techniques, are expected to be increasingly involved in future studies. Multi-omics, encompassing metabolomics, genomics, transcriptomics, proteomics and epigenomics, will provide us with a more comprehensive insight into the biology, diagnostics and therapeutics of sarcopenia [98,99].

## 8. Conclusions

Undoubtedly, sarcopenia in patients with CKD has been linked to reduced QoL. In this review, we analyzed the pathogenetic mechanisms, diagnosis, and therapeutics, referring to investigational drugs as well. With the advent of multi-omics analyses and AI, it seems likely that scientific research will be further expanded regarding sarcopenia in CKD. Clinicians should be aware of sarcopenia and the diagnostic modalities for its early recognition. Nutritional interventions together with regular physical activity should be encouraged in patients with sarcopenia and CKD to further delay its progression. It seems likely that further research will pave the way for a more holistic and personalized approach regarding sarcopenia in patients with CKD.

## Figures and Tables

**Table 1:** Definitions of sarcopenia according to the EWGSOP2 criteria published in 2019.

**Probable** **Sarcopenia**	**Decreased Handgrip Strength or Chair Stand Test**	**Handgrip Strength <27 kg for Males and <16 kg for Females, as Assessed by a Dynamometer, and Chair Stand Test >15 s over 5 Tests**
**Definite Sarcopenia**	Decreased handgrip strength or chair stand test **plus** **low muscle quantity/quality** as assessed by DEXA, BIA, CT or MRI	**ASMM** <20 kg for males and <15 kg for females **ASMI** <7 kg/m^2^ for males and <5.5 kg/m^2^ for females
**Severe Sarcopenia**	Definite Sarcopenia **plus poor physical performance**	A 6 m gait speed ≤0.8 m/s SFPB ≤8 points TUG ≥20 s, i.e., the time to stand up from a chair, walk for 3 m where there is a marker, then turn around, walk back and sit on the same chair.

Abbreviations: ASMI: Appendicular Skeletal Muscle Index; ASMM: Appendicular Skeletal Muscle Mass; SFPB: Short Physical Performance Battery; TUG: Timed up and go test.

**Table 2:** The SARC-F questionnaire.

**STRENGTH**	**Difficulty in Lifting 10 Pounds None: 0 Points**	**Some: 1 Point**	**A lot: 2 Points**
**ASSISTANCE WITH WALKING**	Needs None: 0 points	Some: 1 point	A lot: 2 points
**RISING FROM A CHAIR**	Difficulty None: 0 points	Some: 1 point	A lot: 2 points
**CLIMBING 10 STAIRS**	Difficulty None: 0 points	Some: 1 point	A lot: 2 points
**FALLS**	None: 0 points	Between 1 and 3: 1 points	4 or more: 2 points

## Data Availability

The original contributions presented in this study are included in the article. Further inquiries can be directed to the corresponding author.

## Abbreviations

ASMI: Appendicular Skeletal Muscle Index; ASMM: Appendicular Skeletal Muscle Mass; AI: Artificial Intelligence; AWGS: Asian Working Group for Sarcopenia; BCAAs: Branched-Chain Amino Acids; BIA: Bioelectrical Impedance Analysis; BMI: Body Mass Index; CHARLS: China Health and Retirement Longitudinal Study; CCR: Creatinine to Cystatin C Ratio; CKD: Chronic Kidney Disease; CKM: Cardiovascular–Kidney–Metabolic syndrome; CT: Computed Tomography; CVD: Cardiovascular Disease; DEXA: Dual Energy X-ray Absorptiometry; eGFR: Estimated Glomerular Filtration Rate; ERS: Endoplasmic Reticulum Stress; ESRD: End-Stage Renal Disease; EWGSOP2: European Working Group on Sarcopenia in Older People 2; FGF: Fibroblast Growth Factor; FOXO: Forkhead Box O; FPG: Fasting Plasma Glucose; GDF-15: Growth Differentiation Factor-15; GH: Growth Hormone; GIP: Glucose-dependent Insulinotropic Polypeptide; GLP-1RA: Glucagon-Like Peptide 1 Receptor Agonist; IGF-1: Insulin-like Growth Factor 1; IL: Interleukin; IS: Indoxyl Sulfate; JAK/STAT: Janus Kinase/Signal Transducers and Activators of Transcription Proteins; KDIGO: Kidney Disease: Improving Global Outcomes; LPS: Lipopolysaccharide; MRI: Magnetic Resonance Imaging; mTOR: Mammalian Target of Rapamycin; NF-*k*B: Nuclear Factor Kappa B; NLRP3: Nucleotide-binding Domain-like Receptor Protein 3; Nrf-2: Nuclear Factor Erythroid 2-related Factor-2; PAMPs: Pathogen-Associated Molecular Patterns; pCS: p-Cresyl Sulfate; PEW: Protein Energy Waste; QoL: Quality of Life; RCT: Randomized Controlled Trial; RNS: Reactive Nitrogen Species; ROS: Reactive Oxygen Species; SARC-F: Strength, Assistance with Walking, Rising from the Chair, Climbing Stairs and Falls; SCs: Satellite Cells; SCFAS: Short-Chain Fatty Acids; SFPB: Short Physical Performance Battery; SO: Sarcopenic Obesity; SPPARMα: Selective Peroxisome Proliferator-Activated Receptor-α Modulator; STEP1: Semaglutide Treatment Effect in People with Obesity; TCA: Tricarboxylic Acid; T2DM: Type 2 Diabetes Mellitus; TNF-a: Tumor Necrosis Factor Alpha; TGF-β: Transforming Growth Factor Beta; TJs: Tight Junctions; TIFγ: Transcriptional Intermediary Factor-1γ; TUG: Timed Up and Go Test; TyG Index: Triglyceride–Glucose Index.
